# Pathophysiology of myocardial remodeling in survivors of ST-elevation myocardial infarction revealed by native T1 mapping: inflammation, remote myocardium and prognostic significance

**DOI:** 10.1186/1532-429X-17-S1-Q52

**Published:** 2015-02-03

**Authors:** David Carrick, Caroline Haig, Samuli M Rauhalammi, Nadeem Ahmed, Ify Mordi, Margaret McEntegart, Mark Petrie, Hany Eteiba, Stuart Hood, Stuart Watkins, Mitchell Lindsay, Ahmed Marous, Aleksandra Radjenovic, Ian Ford, Niko Tzemos, Keith G Oldroyd, Colin Berry

**Affiliations:** 1 Golden Jubilee National Hospital, Clydebank, UK; 2 Institute of Cardiovascular and Medical Sciences, University of Glasgow, Glasgow, UK; 3 Robertson Center for Biostatistics, University of Glasgow, Glasgow, UK

## Background

The pathophysiology and prognostic significance of remote myocardium in the natural history of STEMI is uncertain. Cardiac magnetic resonance (CMR) provides a non-invasive assessment of myocardial pathology that is spatially and temporally coordinated. Native T1 quantified by CMR (T1 relaxation time, milliseconds) is a fundamental tissue property determined by water content and cellularity. We aimed to investigate the clinical significance of remote myocardium in survivors of acute ST-elevation myocardial infarction (STEMI) using native T1 mapping.

## Methods

We performed a prospective single center cohort study in reperfused STEMI patients who underwent CMR 2 days and 6 months post-MI and long term follow-up (18 months minimum). Native T1 CMR (MOLLI investigational prototype sequence: 3 (3) 3 (3) 5) was measured in regions-of-interest in remote and injured myocardium. Infarction was depicted on late gadolinium contrast enhancement imaging. Adverse remodeling was defined as an increase in left ventricular end-diastolic volume ≥ 20% at 6 months. Major adverse cardiac events (MACE) were defined as cardiac death or hospitalization for non-fatal MI or heart failure. Results are mean±SD unless specified.

## Results

300 STEMI patients (mean age 59 years, 74% male) gave informed consent (14 July 2011 - 21 November 2012). Of these, 288 STEMI patients had evaluable native T1 CMR and follow-up data (median duration 845 days). Infarct size was 18±14% of left ventricular mass. Two days post-STEMI, native T1 in remote myocardium was lower than native T1 in the infarct zone (961±25 ms vs. 1097±52 ms; p<0.01). In multivariable linear regression, remote zone native T1 was independently associated with incomplete ST-segment resolution (9.42 (2.37 to 16.47); p=0.009), the log of the initial CRP concentration (regression coefficient 3.01 (95% CI 0.016 to 5.55); p=0.038) and the peak monocyte count within 2 days of admission (10.20 (0.74, 19.67); p=0.035).

At 6 months, left ventricular end-diastolic volume increased by 5 (25) ml (n=262 patients with evaluable data) overall, and adverse remodeling occurred in 30 (12%) patients. Remote zone native T1 was a multivariable predictor of the change in left ventricular end-diastolic volume from baseline (0.13 (0.01, 0.24); p=0.035).

39 (13.5%) patients experienced a MACE including 20 (6.9%) patients with a post-discharge MACE. Remote zone native T1 was an independent predictor of post-discharge MACE (hazard ratio 1.016, 95% CI 1.000, 1.032; p=0.048) including after adjustment for changes in LVEF (p=0.032), LV end-diastolic volume (p=0.053), and monocyte count (p=0.036).

## Conclusions

Remote zone tissue characteristics early post-MI are temporally linked with reperfusion injury and inflammation and independently predict left ventricular remodeling and MACE in STEMI survivors.

## Funding

N/A.

**Figure 1:**
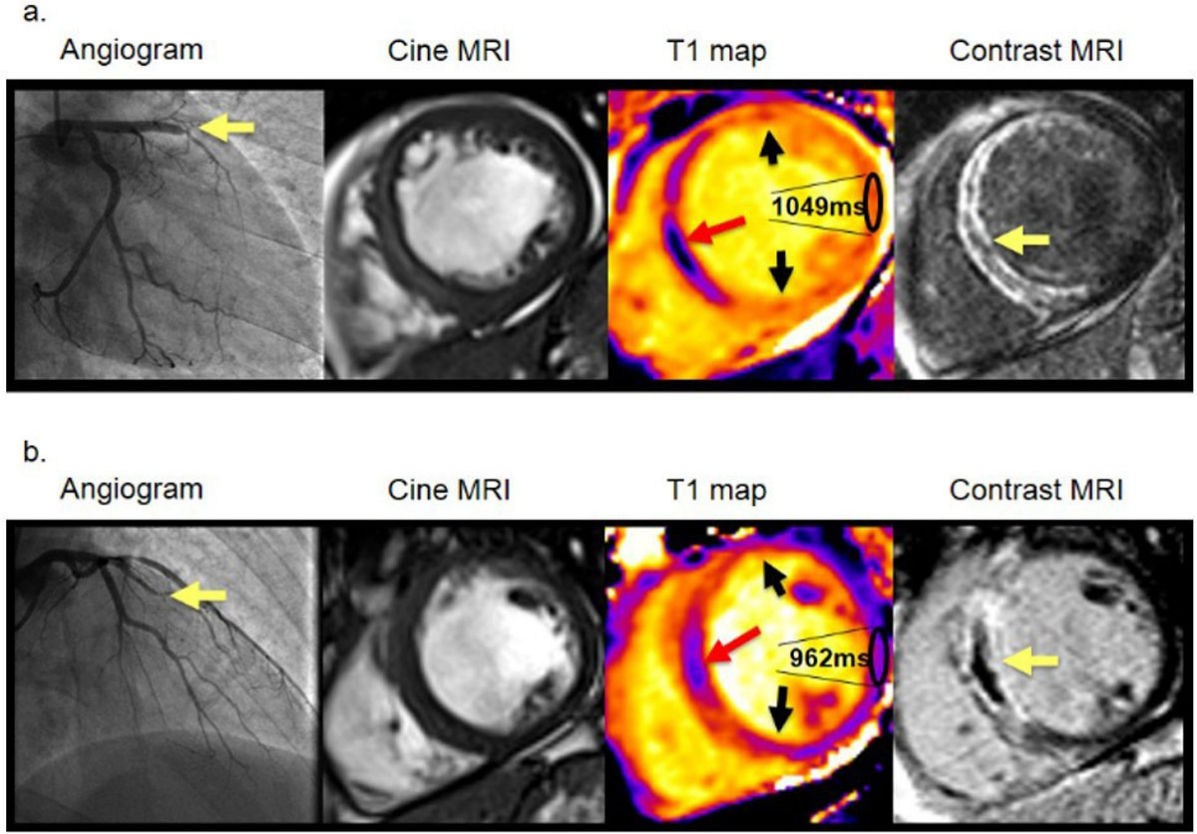
Two patients with acute anterior STEMI treated by primary PCI and with the same standard anti-thrombotic therapies. Each patient had TIMI grade 3 flow at the end of the procedure. (*a*) *Patient with high remote zone native T1.* Six month follow-up MRI revealed final infarct size was 39.2% of left ventricular mass and significant adverse remodeling occurred with left ventricular end-diastolic volume of 145.7 ml/m2. This patient was subsequently hospitalised for new onset heart failure and had an defibrillator device implanted. (*b*) *Patient with average remote zone native T1 value.* The infarct size at 6 months revealed by contrast-enhanced MRI was 31.0% of left ventricular mass and left ventricular end-diastolic volume of 84.3 ml/m2. This patient had an uncomplicated clinical course.

